# Mapping the driving forces of chromosome structure and segregation in *Escherichia coli*

**DOI:** 10.1093/nar/gkt468

**Published:** 2013-06-17

**Authors:** Nathan J. Kuwada, Keith C. Cheveralls, Beth Traxler, Paul A. Wiggins

**Affiliations:** ^1^Department of Physics and Department of Bioengineering, University of Washington, Seattle, WA 98195, USA, ^2^Biophysics Graduate Group, University of California, Berkeley, CA 94720, USA and ^3^Department of Microbiology, University of Washington, Seattle, WA 98195, USA

## Abstract

The mechanism responsible for the accurate partitioning of newly replicated *Escherichia coli* chromosomes into daughter cells remains a mystery. In this article, we use automated cell cycle imaging to quantitatively analyse the cell cycle dynamics of the origin of replication (*oriC*) in hundreds of cells. We exploit the natural stochastic fluctuations of the chromosome structure to map both the spatial and temporal dependence of the motional bias segregating the chromosomes. The observed map is most consistent with force generation by an active mechanism, but one that generates much smaller forces than canonical molecular motors, including those driving eukaryotic chromosome segregation.

## INTRODUCTION

The fitness of all organisms is dependent on the rapid and faithful replication and segregation of the genome to the daughter cells. Although it has long been appreciated that a mitotic spindle drives chromosome segregation in eukaryotic cells, the dominant mechanism exploited by prokaryotic cells is still debated. Active partitioning systems are known to segregate the low-copy-number plasmids (e.g. P1, R1-16 and F) and homologous systems have been found on the chromosomes of *Caulobacter crescentus* and *Bacillus subtilis* and a number of other bacteria (1–7). These active systems are believed to have some functional similarity to spindles but often appear to play a surprisingly limited role: for example, the *par* genes of *B. subtilis* are not essential. Intriguingly, no homologous system has yet been discovered in *Escherichia coli,* and a group of nucleoid structural and segregation genes, including *mukBEF*, *seqA* and *matP*, appear to have supplanted both the bacterial structural maintenance of chromosomes (SMC) and partitioning (*par*) genes in γ-proteobacteria, suggesting that other mechanisms of segregation may play an important role (8,9).

**Figure 1.**
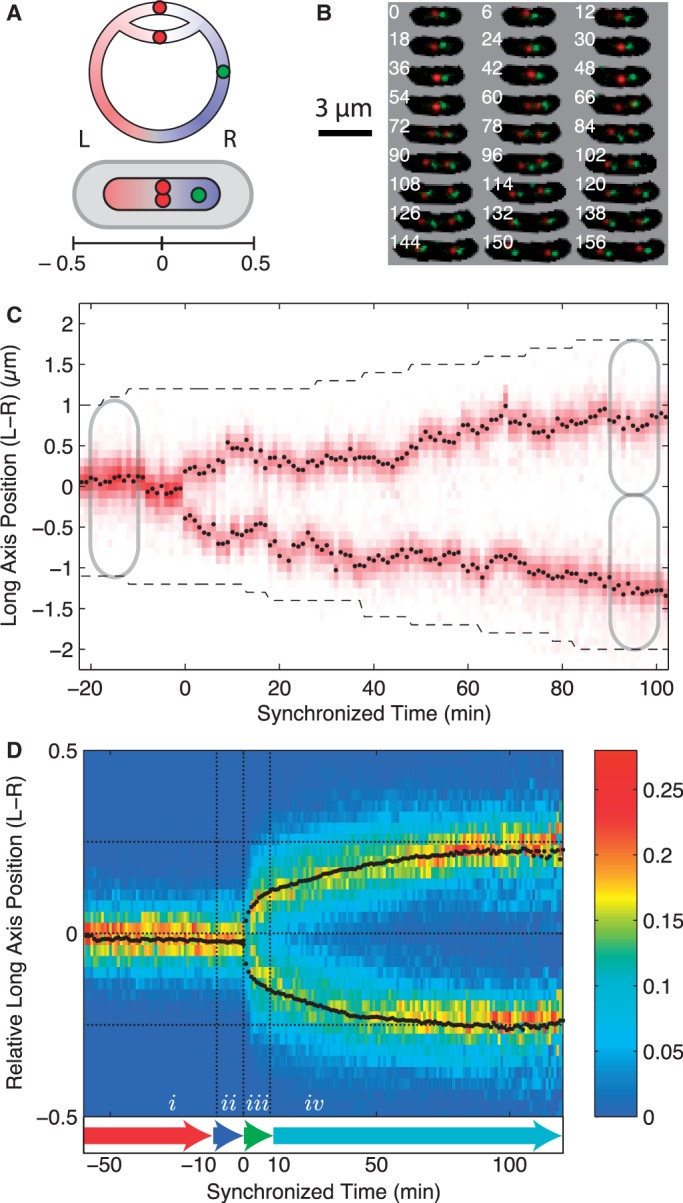
(**A**) Schematic of the bi-directional replicating circular *E. coli* chromosome and the chromosome conformation in the cell. Before the initiation of segregation, *oriC* (red focus) is positioned at mid-cell with the left arm of the chromosome on the left side of the cell and the right arm of the chromosome on the right side of the cell. The chromosomal orientation is measured using the position of a second locus (green focus) on the right arm of the chromosome. The relative long-axis position of foci in the cell is measured relative to cell length from mid-cell. (**B**) Frame mosaic of a typical cell cycle (every 6th frame shown for clarity). An array of phase-contrast/fluorescence composite images shows the red (*oriC*) and green (fiducial) fluorescent foci and the cell mask as a function of time (min) since cell division, which is shown in the top left corner of each image. (**C**) Kymograph for a typical cell shows red fluorescence intensity along the long axis of the cell as a function of time since the splitting of the *oriC* locus. The black points show the fit *oriC* long-axis position of the focus relative to mid-cell as a function of time (min). The black dashed line shows the cell poles. (**D**) Locus position occupancy (heat map) and mean trajectory (black points) of *oriC* as a function of relative cellular position for 406 independent cell cycles synchronized to the *oriC* split (*t* = 0). Dotted horizontal lines show the approximate home positions of the *oriC* locus before and after segregation. Locus dynamics are organized into four time intervals of motion: (i) *Pre-Replication*; (ii) *Cohesion*; (iii) *Rapid-Translocation*; and (iv) *Post-Segregation* for analysis.

Much of what is known about the *E. coli* chromosome segregation mechanism is phenomenological and qualitative: In slow growing cells (generation time ∼120 min), the initial locus dynamics is characterized by a *Stay-at-Home* phenomena where the locus remains localized to mid-cell (10–16). Replication is initiated at the chromosomal origin of replication (*oriC*), and proceeds bi-directionally down the two arms of the circular chromosome (Figure 1Α) (17). After roughly 20 min of cohesion (18), newly replicated sister loci split and undergo *rapid translocation* towards the quarter cell positions (the mid-cell location after division). After reaching the quarter cell positions, *oriC* dynamics is again characterized by a *Stay-at-Home* phenomenon (11,15). In general the rest of the chromosome is replicated and segregated continuously and sequentially, such that genes sequentially closer to *oriC* are replicated and segregated earlier than distant genes (13,18). A number of subtle nucleoid structural transitions have also been reported (T1, T2 and T3), in which loci on the right arm of the chromosome split cooperatively (19,20).

In this article, we perform a quantitative analysis of the motion of *oriC*, one of the first loci to segregate (16,19). By combining time-lapse epi-fluorescence microscopy with high-throughput automated image analysis, we are able to capture *oriC* dynamics throughout the cell cycle for greater than an order-of-magnitude more cells than have ever been characterized. This collection of complete cell cycle trajectories facilitates the quantitative analysis of the locus motion summarized qualitatively above. We report the following findings: (i) Mean-Squared Displacement (MSD) analysis of the *Rapid-Translocation* phase of *oriC* motion shows sub-diffusive dynamics, rather than processive dynamics. (ii) Similar dynamics are observed for the actively partitioned plasmid R1-16 by MSD analysis, demonstrating that processive dynamics on times scales shorter than a cell cycle are not a prerequisite for active segregation mechanisms. (iii) A comparison of the step-size distribution between the *Rapid-Translocation* and *Stay-at-Home* phases of locus motion shows a distribution-wide bias towards the eventual destination, rather than the presence of large biased steps. (iv) Faithful segregation of the origin loci results from a small diffusional bias, a drift velocity, that switches from a restoring force, centred around mid-cell before locus segregation, to a restoring force centred around the quarter cell positions immediately proceeding locus splitting. The cell appears to identify the quarter cell positions in advance of the arrival of *oriC* suggesting the existence of a cellular landmark determining this position. Because the nucleoid is significantly remodelled during this period while the drift velocity remains qualitatively unchanged, it is unlikely that nucleoid structure (19,20) or chromosome entropy (21) is the dominant source of the diffusional bias and therefore suggests the existence of an additional as-yet undiscovered segregation mechanism in *E. coli*. The measurement of the drift velocity and the interpretation of this velocity in terms of a driving force provide the first clear biophysical picture of the dynamical changes that drive the segregation process and reconcile the seemingly conflicting observations of sub-diffusive MSD scaling and active segregation. We expect this analysis to be applicable not only to the interpretation of other chromosome dynamics problems, but also to sub-cellular stochastic motion in general.

## MATERIALS AND METHODS

### Strains and growth conditions

To image chromosomal loci during replication, we incorporate both the Fluorescent Repressor Operator System (FROS) and the ParB-*parS* system to label two distinct regions of the chromosome simultaneously. All strains are derivative of AB1157 (22) with a lac sequence 15 kb counter clockwise from *oriC* transduced from IL06 and a *parS* cassette (used for left–right spatial orientation) inserted at the endogenous *lac* locus (23). Each strain was transformed with an expression plasmid (pNT1) under the *araC* promoter with both GFP-ParB and mCherry-lacI fusions (see Supplementary Material*, Sec. 6*). Strains were grown in M9 minimal media supplemented with 0.2% glycerol, 100 μg/ml of arginine, histidine, leucine, threonine, proline and 10 μg/ml thiamine HCl. Prior to imaging, cells were induced with 0.02% L-arabinose for 30 min at 30°C. The generation time of cells was ∼120 min.

### Microscopy

Time-lapse phase-contrast and wide-field fluorescence microscopy images were collected once per minute for 6–8 h using a Nikon Ti-E inverted epi-fluorescence microscope outfitted an environmental chamber. Agarose pads were prepared by pouring 1 ml of growth media with 0.2% agarose into 2 cm × 2 cm wells cut into a rubber gasket sealed onto a standard microscope slide. 2 μl of media containing mid-log cells at OD_600_∼0.1 were spotted onto dried pads and a coverslip was placed on top of the pad. The entire slide was sealed with VALP (1:1:1 Vaseline, lanolin, paraffin) and the pads were allowed to equilibrate at 30 C for 1 h before imaging. Automated image acquisition was controlled by NIS-Elements using an Andor Neo CMOS camera.

### Image analysis

Cells were identified and linked frame-to-frame using automated custom MATLAB segmentation software. To isolate chromosome motion that is independent of cell growth, we express loci coordinates in terms of a fraction of cell length. For complete cell cycle analysis, we only include trajectories that only have one splitting event, i.e. one focus at the beginning of the cell cycle and two foci when the cell divides. The full collection of trajectories used for analysis is comprised of data from multiple experiments, and are freely available in the Supplementary Material*.*

## RESULTS

### Mean locus trajectories

To quantitatively analyse the chromosome segregation process, we visualized and tracked the physical location of fluorescently labelled *oriC* throughout the cell cycle and characterized the changes to the dynamics between the *Stay-at-Home* and *Rapid-Translocation* phase of *oriC* locus motion. The labelling scheme is shown schematically in Figure 1A, a frame mosaic of a complete cell cycle is shown in Figure 1B, and a typical kymograph showing the dynamics of long-axis fluorescence for a single cell cycle is shown in Figure 1C. The kymograph clearly illustrates the stochastic nature of locus motion and the necessity of using a statistical approach on a large ensemble of trajectories to accurately quantify the motion.

To average or compare dynamics between cells, *oriC* trajectories are synchronized to the splitting of the *oriC* locus into two distinct fluorescent foci. [Note that due to sister cohesion following replication, this splitting event does not correspond to the replication time, but roughly 20 min after the locus has been replicated (19,20,24)]. To account for the existence of asymmetries in the segregation pathway, we use a second fiducial marker on the chromosome to orient cells by the *left–right* spatial orientation of the chromosome (L–R), and in order to isolate locus motion that is independent of cellular growth, we express the long-axis *oriC* position as a fraction of cell length relative to mid-cell. (See the axis defined in Figure 1A. Full trajectory files are included in the Supplementary Material.)

The mean long-axis locus trajectory (and the distribution of locus positions) synchronized to the *oriC* split (*t* = 0 min) is shown for 406 independent cells in Figure 1D. We see that *oriC* undergoes a simple segregation program: Both the mean trajectory and the locus distribution function show a rapid initial movement towards the quarter cell positions with a smooth, asymptotic approach over the remainder of the cell cycle. Because the occupancy distribution for each locus has single maximum at each time point and there is such close agreement between the distribution maxima and the mean, there does not appear to be several distinct segregation pathways. Furthermore, the unimodal structure of the distribution also argues against the existence of an asymmetric segregation pathway with respect to the left–right nucleoid orientation (e.g. new-pole versus old-pole).

The rapid initial movement after the splitting of *oriC* would seem to suggest that the dynamics after the locus splitting may be active and processive followed by dynamics dominated by random motion. In order to investigate changes to the dynamics between the *Stay-at-Home* and *Rapid-Translocation* intervals of motion, we divided the segregation process into four characteristic time intervals for further analysis: (i) *Pre-Replication* (*t* < −10 min); (ii) *Cohesion* (−10 min < *t* < 0 min); (iii) *Rapid-Translocation* (0 min < *t* < 10 min); and (iv) *Post-Segregation* (10 min < *t*), where *t* = 0 min is the *oriC* split. These intervals are shown schematically in Figure 1D and were chosen to *roughly* correspond to the qualitative description of the segregation model described in the Introduction. The qualitative conclusions drawn from the analysis do not depend on the precise definition of these four intervals. The 10-minute durations of the *Cohesion* and *Rapid-Translocation* time intervals were chosen to allow the locus dynamics to be as distinct as possible from the rest of the cell cycle while maintaining a long enough trajectory for analysis.

### Mean-squared displacement

To determine whether the motion in the *Rapid-Translocation* interval is processive, we apply the canonical mean square displacement (MSD) analysis. The MSD can be approximated as a power law

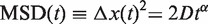

where the dynamics are characterized by two parameters: *α* is referred to as the *scaling parameter* and *D* is the generalized diffusion constant (25,26). Processive motion (positive correlation between successive steps) is characterized by *α* ∼ 2, diffusive motion (no correlation between successive steps) is characterized by *α* ∼ 1, and sub-diffusive motion (anti-correlation between successive steps (There are additional phenomena that can lead to sub-diffusive motion, but this mechanism is the most applicable to *in vivo* loci dynamics.)) by *α* < 1. 

**Figure 2.**
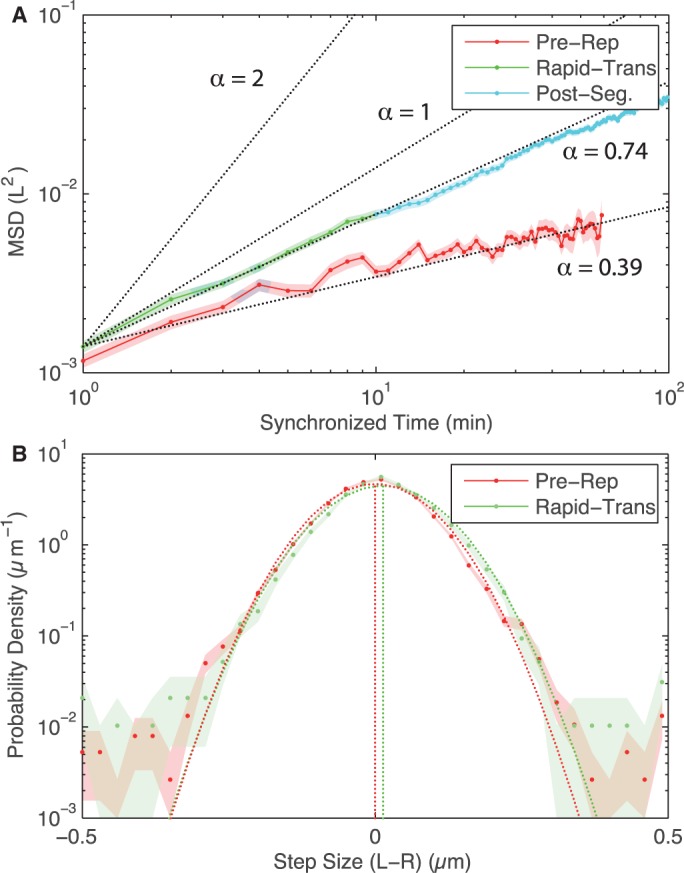
(**A**) MSD for *oriC* prior to and post-loci splitting both show sub-diffusive motion. In the *Pre-Replication* interval of motion, the MSD scaling parameter *α* is 0.39. After *oriC* splits, *α* is 0.74 during the *Rapid-Translocation* and *Post-Segregation* intervals of motion. Even immediately after the initial locus split, *oriC* dynamics is characterized by a scaling factor considerably smaller than *α* = 2 which corresponds to processive motion. (**B**) The step-size distributions (over 1 min) for *oriC* for the *Pre-Replication* and *Rapid-Translocation* intervals of motion. (In order to capture the bias, we consider only the right moving locus after the split. Other intervals are omitted for clarity.) For each distribution, a Gaussian distribution with the same mean (vertical dotted line) and variance is plotted (dotted curve), representing the step-size distribution for a diffusive model. Both intervals of motion have less than 0.5% more large steps than the diffusive step-size distribution. Biased motion during the *Rapid-Translocation* interval is the result of a distribution-wide shift to rightward steps rather than a small number of large steps forward biased steps. (Shaded regions represent standard error).

A number of groups have already measured MSD scaling parameters for *E. coli* chromosomal loci in the non-replicating phase (27,28) and segregating loci on short-time scales (29), reporting *α* = 0.39 and 0.6 respectively, but these studies do not systematically analyse the change in dynamics as a function of segregation phase. Our hypothesis is that the period of processive motion may be quite short and we therefore want to apply the MSD analysis during the initial phase of the segregation process. Our complete cell cycle trajectories facilitate the measurement of MSD in the *Stay-at-Home* and *Rapid-Translocation* (Due to difficulty in determining the exact split time, we begin our MDS analysis at the first point where two distinct foci can be determined, defined as *t* = 1 min in the synchronized tracks, therefore our results do not include dynamics during the minute immediately following splitting.) intervals of motion independently. In the *Pre-Replication* interval, locus trajectories are characterized by a scaling parameter of *α* = 0.38 ± 0.01 and the *Rapid-Translocation* and *Post-Segregation* intervals are characterized by a scaling parameter of *α* = 0.74 ± 0.01 (Figure 2A), in rough agreement with previous short-time scale measurements (27–29). Even immediately following the splitting of the *oriC* locus the scaling parameter is clearly significantly smaller than 2, suggesting that if processive motion does occur, it must happen on times scales shorter than 2 min after locus separation. The motion of *oriC* appears to be dominated by sub-diffusive motion rather than highly processive, spindle-like motion throughout the cell cycle.

### Active plasmid segregation

The sub-diffusive motion of the chromosomal loci during segregation would appear to imply a passive segregation model. As a biological control, we analysed a plasmid system that is known to be actively segregated. The low-copy number plasmid R1-16 incorporates a Type II plasmid partitioning system in which dynamically polymerizing ParM filaments, whose growth is stabilized by associating with a specific sequence on the plasmid, push plasmid copies away from each other to opposite ends of the cell (1,2). MSD analysis of R1-16 plasmid trajectories, shown in detail in Supplementary Material*, Sec. 3*, is characterized by a scaling parameter of *α* = 0.56 ± 0.04 in both the pre- and post-split phases. This surprising result, discussed further in Supplementary Material*,* does not imply that R1-16 partitioning is a passive process, but rather demonstrates that an active segregation mechanism can still exhibit diffusive-like dynamics across the finite time scale of the cell cycle. Because of the biologically limited time scale of the cell cycle, it is clear that a scaling parameter fit in an MSD analysis does not reliably determine whether chromosomes and plasmids are actively segregated.

### Step-size distributions

Even though the MSD is dominated by sub-diffusive motion, chromosome dynamics are strongly biased on the timescales of a cell cycle to produce equal partitioning between daughter cells. The quantitative nature of this bias is not yet understood. It has been reported that rapid unsnapping-transitions result in large steps in locus position that drive the motion of the loci during the segregation process (20). To investigate the possibility that a small number of large biased steps drive the segregation process, we analysed the step-size distribution for locus displacement between frames (for a frame rate of 1 min) for each interval of locus motion and compared this to a Gaussian step-size distribution predicted by a simple diffusion model. If segregation were driven by large biased steps, we propose that we would observe a significant number of these steps in the *Rapid-Translocation* interval as opposed to the *Pre-Replication* intervals of segregation. These distributions are shown in Figure 2B, and three lines of evidence present in the distributions refute this proposal. What is initially striking is not that there appear to be significant differences between the step-size distributions, but instead how similar and Gaussian the step-size distributions are. All intervals of segregation exhibit a higher probability of large steps than predicted by diffusive motion, perhaps consistent with the large-scale nucleoid reorganization events previously reported (20). But, these large steps are not unique to the *Rapid-Translocation* interval. In addition, during the *Rapid-Translocation* interval these large steps occur in just less than 5% of cells, and occur in both the forward and reverse directions. Furthermore, these large steps need not be well synchronized with the *oriC* split to be observed by analysis of the step-size distribution. We have also performed these same analyses for larger time intervals, shown in Supplementary Material*, Sec. 2*, and found the same results. These observations all suggest that large biased steps of *oriC* are not the responsible for biased motion in segregation dynamics.

### Spatiotemporal drift velocity profile

A careful comparison of the step-size distributions between the *Rapid-Translocation* and *Pre-Replication* intervals of motion reveals that there is a small distribution-wide bias for forward versus reverse step direction during the *Rapid-Translocation* interval, consistent with a combination of diffusion with a drift velocity (The mean step-size is represented by the vertical dotted lines in Figure 2B). This shift in the mean is also seen in the segregation dynamics of both *Vibrio cholera* chromosomes (30). We interpret the mean step-size as a drift velocity

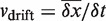

where δ*x* is the difference is relative position δ*t* is the time between frames (1 min). The variance of the step-size is interpreted as the effective diffusion constant

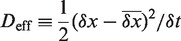



**Figure 3.**
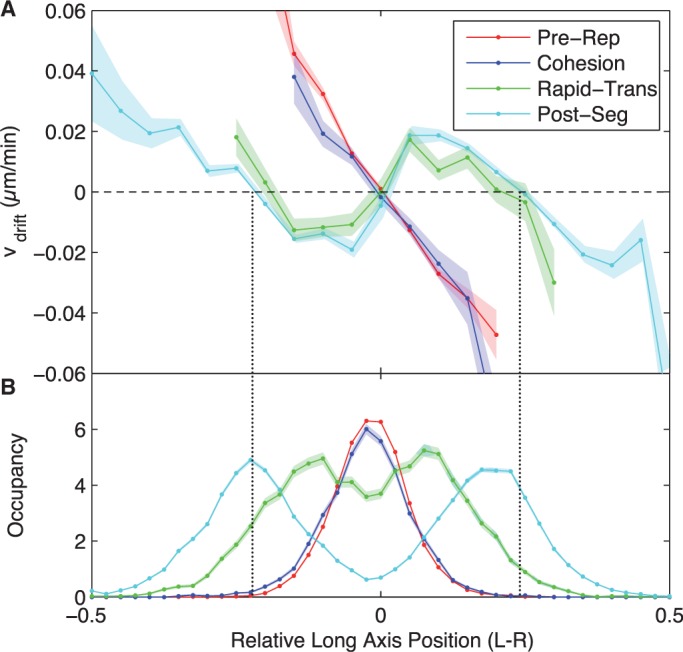
(**A**) Spatiotemporal dependence of the drift velocity of *oriC*. During the *Pre-Replication* and *cohesion* intervals of locus motion, there is a restoring drift velocity to the equilibrium position of the locus at mid-cell. Immediately after the *oriC* loci split, the mid-cell position becomes unstable and equilibrium positions 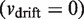 appear at the quarter cell positions. This velocity profile remains qualitatively unchanged for the remainder of the cell cycle. (**B**) Spatiotemporal dependence of locus occupancy. Higher mean velocity is observed in the *Rapid-Translocation* interval than in the *Post-Segregation* interval of motion since the peak occupancies (maxima of the occupancy curves) are further from the equilibrium positions (vertical dotted lines). (Shaded regions represent standard error).

The drift velocity as a function of segregation interval and relative cellular position is shown in Figure 3A. Before the *oriC* split, there is a restorative drift velocity profile that returns *oriC* to mid-cell. For instance, *oriC* loci to the right (*x* > 0) of mid-cell have a negative drift velocity that moves them back towards mid-cell (*x* = 0) on average. The restoring velocity is approximately linear in the displacement of the locus from the equilibrium position, reminiscent of a damped linear spring.

Immediately after the split and for the duration of the cell cycle, mid-cell localization becomes unstable and the equilibrium positions are shifted to the quarter cell positions (Figure 3B). Surprisingly, this drift velocity profile changes little during the remainder of the cell cycle, despite the assembly of the sister nucleoids around the origin as the cell cycle progresses (13,20,31). The effective diffusion constant and visco-elastic memory (autocorrelation between successive steps) are analysed in the Supplementary Material*, Sec. 4*. *oriC* shows a slight increase in the effective diffusion constant (50%) during the *cohesion* and *Rapid-Translocation* intervals of segregation and a decrease in visco-elasticity, consistent with the increase of the scaling parameter in our MSD analysis.

### Langevin interpretation

To interpret the drift velocity, it is convenient to approximate the coarse-grained motion of *oriC* with the overdamped Langevin equation,

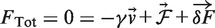

where the viscous force 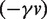 balances the applied forces. The applied force, which could result from interactions with the replicating nucleoid, thermal fluctuations, a putative spindle, transient membrane attachments, etc., is divided into a fluctuating part 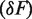, which is zero on average and is responsible for diffusive motion, and a time-averaged force (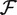). In this model, the drift velocity is determined by ensemble averaging the Langevin equation:

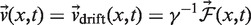

where γ is the viscous drag coefficient, which we assume is roughly constant throughout. The drift velocity can therefore be interpreted as roughly proportional to the force responsible for biasing *oriC* motion (It should be noted that this simple model is not sufficient to recreate sub-diffusive dynamics, but because we are specifically interested in dynamics unrelated to stochastic thermal motion, e.g. drift velocity, it is an appropriate approximation for our current analysis.).

**Figure 4.**
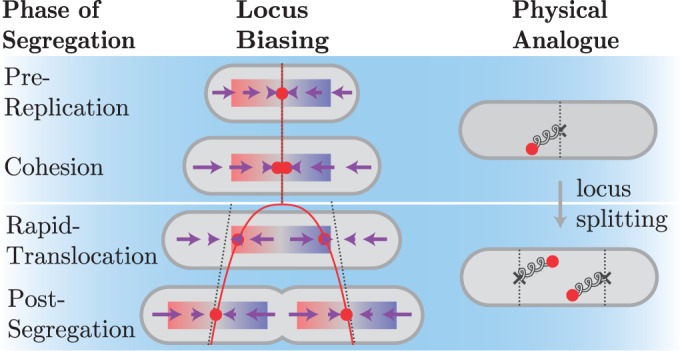
Schematic model of spatiotemporal drift velocity profile for *oriC*. During both the *Pre-Replication* and *Cohesion* intervals, there is a restoring force (purple arrows) to the equilibrium position (dotted line) at mid-cell. This restoring force is represented by a spring in the physical analogue connecting the loci to mid-cell. Immediately upon *oriC* locus separation, the equilibrium position moves to quarter cell (dotted lines) and remains qualitatively unchanged for the remainder of the cell cycle, despite significant changes to the nucleoid structure. In the physical analogue system, a spring connects each locus to the quarter cell position. The mean locus velocity is initially high (*Rapid-Translocation*) since the locus begins far from the equilibrium position (dotted line). Once the locus is close to the equilibrium position, the force is low, corresponding to the *Post-Segregation* interval of motion.

During the *Pre-Replication* and *Cohesion* intervals of the cell cycle, the drift velocity profile is most consistent with a Hookean restoring force (linear in displacement) towards mid-cell, shown schematically in Figure 4. This is also consistent with our previously proposed elastic filament model of the non-replicating chromosome (23). The slope of 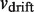 with respect to long-axis position gives the relaxation time’, τ ∼10 min, which explains why the MSD curve is sub-diffusive rather than saturated in previous studies that probed shorter time scales (27,29). Immediately following *oriC* splitting, and for the remainder of the cell cycle, the drift velocity is consistent with two Hookean spring potentials centred about the quarter cell positions, which eventually become the new cell centres of the daughter cells following cytokinesis (Figure 4). The rapid movement of these equilibrium positions imply that *oriC* can recognize its equilibrium position and reverse its direction if it travels too far. These results have interesting implications for the mechanism of *oriC* segregation.

### In search of the centromere

A putative centromeric sequence has been proposed for *E. coli*: *migS*, a 25 bp sequence within the non-essential gene *yijF*, was reported to affect bi-polar positioning of *oriC* following replication (32,33). To investigate whether this sequence was required to generate the observed diffusive bias, we deleted the *migS* sequence and analysed the motion of *oriC*. The spatiotemporal drift velocity profile for 341 cells is shown in Supplementary Material*, Sec. 5*. The drift velocity profile is qualitatively unchanged and in particular retains the rapid shift in equilibria position to the cell quarters immediately after the splitting event. The *migS* sequence is therefore not required for the generation of diffusive bias of the origin in the segregation process. It is straightforward to expand this quantitative analysis of diffusive bias to other deletions.

## DISCUSSION

### Mechanisms of segregation

Many segregation mechanisms have been proposed for the *E. coli* chromosome (34). One of the first proposals was that the newly replicated chromosome loci attached to the growing cell membrane (35). As has been reported before, we see from the mean *oriC* trajectory (Figure 1C) that locus movement during segregation is much faster than the elongation of the cell, ruling out the membrane tethering model (36). A number of models propose that DNA synthesis during the replication process, and the resulting forces due to DNA excluded volume, drive chromosome segregation (17). In the *replication factory* model*,* stationary DNA-replication machinery extrudes daughter chromosomes (37). It is assumed that there is no mixing between sisters or between newly replicated and unreplicated DNA, such that force is generated by the excluded volume of the chromosomes and the build-up of newly replicated DNA. The entropic demixing model attempts to explain the failure of the chromosomes to mix as a consequence of the polymer entropy and excluded volume (21,38,39). In both the *replication factory* model and the demixing model, nucleoid structure generates the forcing. Since the nucleoid undergoes significant remodelling and large-scale structural changes during the replication process, we would predict the force profile that results from this structure to significantly change between the initial splitting of the *oriC* and the end of the cell cycle. This is not observed in the drift velocity profile. For instance, in the replication factory model would predict that the equilibrium position of *oriC* would move slowly outwards from mid-cell with the accumulation of the newly replicated DNA. This prediction does not seem to be consistent with the immediate movement of the equilibrium position of the origin to the quarter cell positions after locus splitting. (It should be noted that Figure 3A shows a small shift outward of the equilibrium position during *Post-Segregation* interval, perhaps consistent with the accumulation of newly replicated DNA or other changes in the chromosome structure.) In fact, the static forcing profile of *oriC* is consistent with the same mechanism being responsible for both structural maintenance and segregation.

Another set of recent studies of the *E. coli* segregation process have reported nucleoid structural transitions during the segregation process which are proposed to represent the loss of sister cohesion over large regions of the chromosome (19,20). Our investigations have failed to find a clear signature of these rearrangements in the movement of *oriC* after the initial splitting event in either the analysis of the mean locus trajectories or in the step-size distribution, suggesting while these structural rearrangements may play a significant role in some strains, they do not seem to be an essential element in *E. coli oriC* segregation in all strains.

An active spindle-like mechanism could well generate the drift velocity profile observed. We can make an order-of-magnitude estimate of the force required to generate the observed bias as follows: If we assume that the observed fluctuations are not significantly larger than thermal fluctuations (40), we can use the Einstein Relation to roughly estimate the viscous drag from the effective diffusion coefficient:

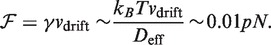

This force scale is substantially smaller than canonical eukaryotic molecular motors, but such a small force could theoretically be generated by *oriC*-bound proteins interacting with an actively established protein gradient (41,42).

## CONCLUSIONS

The quantitative approach presented in this article to the characterization of the motion of genetic loci during the segregation process demonstrates the limits of MSD-based analyses and presents a new alternative: the measurement of the drift velocity. This measurement for the first time presents a clear signature of the microscopic bias that drives *oriC* segregation and is consistent with the existence of an as-yet undiscovered mechanism of segregation in *E. coli*.

## SUPPLEMENTARY DATA

Supplementary Data are available at NAR Online: Supplementary Figures 1–11, Supplementary Methods, Supplementary Analysis and Supplementary Datasets [1–6].

## FUNDING

National Science Foundation [PHY-084845, MCB-1151043]; Alfred P. Sloan Foundation; University of Washington Royalty Research Fund. Funding for open access charge: The National Science Foundation.

*Conflict of interest statement*. None declared.

## Supplementary Material

Supplementary Data
