# Supplementary material for: Mapping the driving forces of chromosome structure and segregation in Escherichia coli
Source: Nucleic Acids Res. 2013 Jun 17;41(15):7370–7. doi: 10.1093/nar/gkt468 (PMC3753618; doi:10.1093/nar/gkt468)
Supplement: Supplementary Data [file supp_41_15_7370__index.html]

Mapping the driving forces of chromosome structure and segregation in Escherichia coli — Mapping the driving forces of chromosome structure and segregation in Escherichia coli — Supplementary Data 

# Mapping the driving forces of chromosome structure and segregation in *Escherichia coli*

## Supplementary Data

files

**Files in this Data Supplement:**

- Supplementary Data - zip file
