# Supplementary material for: Mapping the driving forces of chromosome structure and segregation in Escherichia coli
Source: Nucleic Acids Res. 2013 Jun 17;41(15):7370–7. doi: 10.1093/nar/gkt468 (PMC3753618; doi:10.1093/nar/gkt468)
Supplement: Supplementary Data [file supp_gkt468_nar-00959-h-2013-File006.zip › seg_paper_nar_supplemental_resub/Dataset Legend.rtf]

oriC Trajectories from Kuwada, et al. "Direct measurement of diffusional bias of the origin of replication of Escherichia coli"Corresponding author: Nathan J. Kuwada, nateman@uw.eduList of files:"Synchronized Trajectory Files"Dataset 1: oriC_synch_length.txtDataset 2: oriC_synch_down.txtDataset 3: oriC_synch_up.txt"Split Trajectory Files"Dataset 4: oriC_split_length_presplit.txt	Dataset 5: oriC_split_length_postsplit.txtDataset 6: oriC_split_presplit.txtDataset 7: oriC_split_postsplit_down.txt	Dataset 8: oriC_split_postsplit_up.txt	- All coordinates are represented as fraction of cell length.  The cell length as a function of time for each trajectory is included for both the synchronized tracks and split tracks for conversions.  - The synchronized trajectories are constructed to have the split at t = 200.  They are split into the upper and lower tracks, but they both include the same data for the pre-splitting region.  - The split tracks are arranged as pre-split and upper and lower post split tracks. 
