# Supplementary material for: Mapping the driving forces of chromosome structure and segregation in Escherichia coli
Source: Nucleic Acids Res. 2013 Jun 17;41(15):7370–7. doi: 10.1093/nar/gkt468 (PMC3753618; doi:10.1093/nar/gkt468)
Supplement: Supplementary Data [file supp_gkt468_nar-00959-h-2013-File006.zip › seg_paper_nar_supplemental_resub/NJK_supplemental_material_NAR.pdf]

Supplemental Material for *Mapping the forces driving chromosome structure and segregation in Escherichia coli*

Nathan J. Kuwada and Paul A. Wiggins
Department of Physics and Department of Bioengineering, University of Washington, Seattle, WA, 98195

Keith C. Cheveralls
Department of Molecular and Cellular Biology, University of California, Berkeley, Berkeley, CA, 94720

Beth Traxler
Department of Microbiology, University of Washington, Seattle, WA, 98195
(Dated: April 30, 2013)

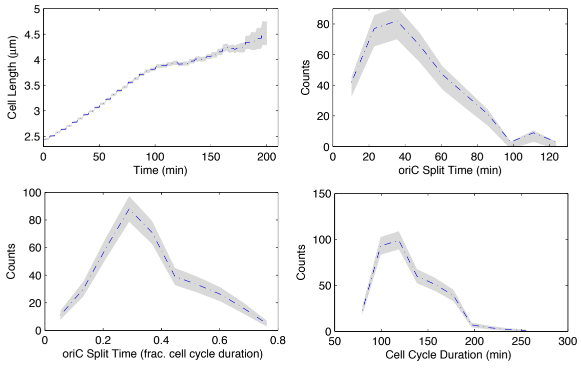

FIG. S1. Examples of ensemble measurements of cell cycle-specific characteristics: (A) Average cell length as a function of time, (B) distribution of *oriC* splitting time, (C) *oriC* splitting time as a function of cell cycle duration, and (D) distribution of cell cycle duration.

S1. ADDITIONAL CELL CYCLE STATISTICS

From our data we are able to construct a large set of statistical measurements that are contingent on a large data set of complete cell cycles. For example, Fig. S1 shows the mean cell length as a function of time, the mean splitting time of *oriC* in absolute time and relative to the cell cycle, and the mean cell cycle length. These examples are a small subset of the analysis now possible with current available technology.

S2. LARGE STEPS SYNCHRONIZED TO SPLITTING RATHER THAN REPLICATION

It has been reported that there exist a small number of large scale steps during the segregation process of *oriC* as a result of abrupt unsnapping of specific cohered regions of the chromosome copies (19,20). These unsnapping events are thought to be well coordinated to *oriC* replication. Since our tracks are synchronized by the locus splitting time rather than replication, we wanted to explore whether or not these large scales steps are also coordinated to *oriC* splitting.

Fluctuations about the Mean Trajectory

Features in the mean trajectory shown in the main text appear smooth. In order to better quantify the smoothness of the trajectory we calculate the relative error, which we define as the ratio of the trajectory fluctuations about the local mean compared to the error,

$$RE(t) = \frac{x(t) - \overline{x}_{\text{local}}}{\text{error}(t)} \ , \tag{1}$$

where the local mean, $\overline{x}$ is calculated using the standard MATLAB gaussian filter (hsize = 20, standard deviation = 5). The relative error provides a way to determine the significance of apparent features in the trajectory. If there are any large scale structural rearrangements of the chromosome that are well synchronized to the splitting of *oriC*, we would expect to observe well defined peaks in the relative error signifying trajectory transitions that are significant compared to the error. The relative error for 60 minutes following *oriC* splitting is shown in Fig. S2. Besides the *Rapid-Translocation* phase (up to t = 10 min), where we expect the motion to be larger the error, we observe no other regions in which the fluctuations about the mean are greater than the error. This result does not preclude the existence of large scale steps in the trajectory, but it suggests that if these transitions exist they are not precisely synchronized to *oriC* splitting.

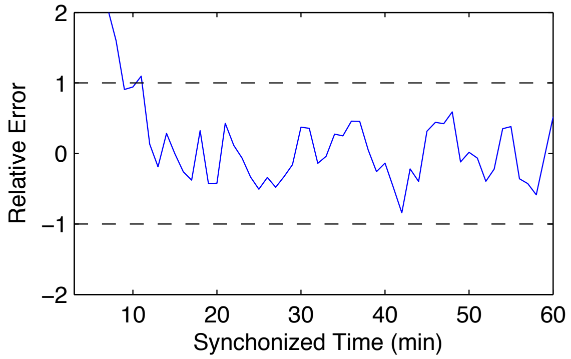

FIG. S2. Relative error of mean *oriC* trajectory for 60 minutes following splitting. Large steps in the individual trajectories that are well synchronized to the splitting time would manifest as large steps in the relative error of the mean track. Besides the *Rapid-Translocation* phase (t<10 mins), we observe no other occurrences of large, significant features in the mean trajectory, suggesting the absence of large scale steps well synchronized to *oriC* splitting.

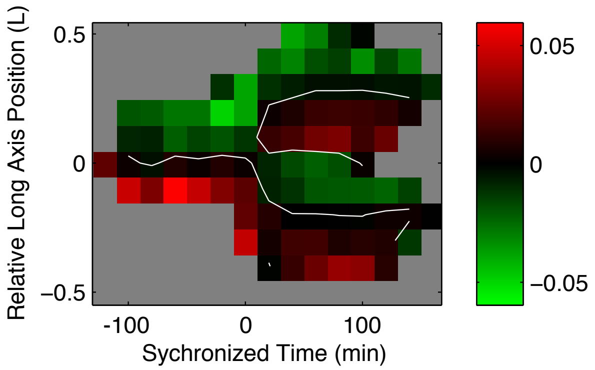

FIG. S3. Spatiotemporal velocity distribution with $v_{\rm drift} = 0$ overlaid in white, where $v_{\rm drift}$ is determined by the mean velocity across 10 minute intervals. Red represents $v_{\rm drift} > 0$ and green $v_{\rm drift} < 0$. As with the mean velocity profile, we observe a rapid shift in the $v_{\rm drift} = 0$ (signifying zero net force) to the quarter cell positions following separation. We do not observe any features that signify large steps that are well synchronized to *oriC* splitting.

Spatiotemporal Drift Velocity Distribution

In the main text we show the mean spatiotemporal drift velocity profile, which does not give any indication of large, synchronized steps after the *Rapid-Translocation* phase, although this result does not necessarily preclude these events. Instead of looking at the mean, we can also look for features in the distribution of drift velocity. Figure S3 shows the drift velocity as a function of space and time for 10 minute time intervals with the $v_{\rm drift} = 0$ contour overlaid for clarity. We observe no features in the distribution or the contour that would suggest events that occur on order 10 minutes.

Step Size Distribution for Longer Time Scales

Because we orient our trajectories spatially rather than by locus dynamics, if there exist a significant difference in the motion of the sister loci, e.g. one translocating a longer distance than the other, it should manifest itself as a bimodal step-size distribution function. We do not see this in the single step distributions (interval 1 minute) presented in the main text, so we decided to increase the step size interval to 10 minutes to look for features that occur on longer time scales, shown in Fig. S4 for only the rightward (positive) moving locus. We recreate the shift in

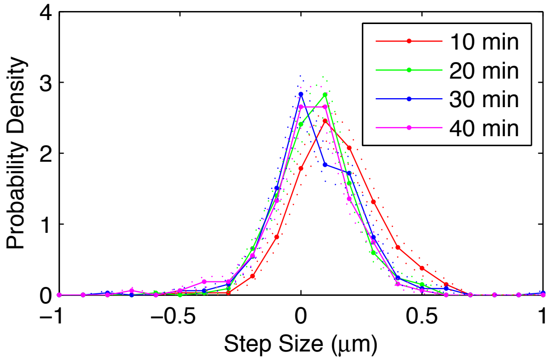

FIG. S4. Step size distributions for 10 minute intervals, e.g. t = 1-10, 10-20, etc., where the legend refers to the final time point of the interval. We still observe the shift in the distribution during the *Rapid Translocation* phase (10 min). Characteristic differences in sister loci motion on this time scale would produce a bi-modal distribution, which we do not observe.

the longer time interval distributions during the *Rapid Translocation* phase as we saw before, but we do not observe a bimodal distribution in any 10 minute time period following separation.

Dynamics Synchronized to Cell Cycle

For the analyses in the main text, as well as the discussion above, we pool together *oriC* trajectories from cells with a distribution of cell cycle lengths (Fig. S1(D)) in order to increase our statistics. Because of this, if large scale steps are synchronized to both *oriC* splitting *and* specific checkpoints in the cell cycle, these events would likely appear stochastic in our data and be averaged out. In an effort to explore this potential problem, we isolated trajectories from our data whose cell cycle was between 115-125 minutes (158 cells). Figure S5 shows a similar step size distribution to the full data set, i.e. a slight shift in the mean step size (indicative of a drift velocity) and a slightly higher prevalence of large steps during the *Rapid-translocation* phase, consistent with a slightly higher mobility (Fig. S9), but these large steps appear bi-directional and thus likely are not completely responsible for faithful *oriC* segregation. Furthermore, the spatiotemporal drift velocity profile shown Fig. S6 matches the full data set very well, suggesting mechanism responsible for the drift velocity is not necessarily tightly coupled to the cell cycle.

It should be noted that due to the fact we observe a broad distribution of *oriC* splitting time relative to cell cycle duration (Fig. S1(C)), large steps coordinated to both replication and the cell cycle may still appear stochastic when trajectories are synchronized to the distribution of splitting events. The only way to observe steps of this nature in our current analysis would be to only consider an even smaller subset of cells with similar cell cycle duration *and* splitting time, of which we currently do not have enough to produce statistically significant results.

Strain Differences

Although these results are consistent with previous investigations of AB1157 (the strain used in this study) (22), they show significant differences with investigations of other *E. coli* strains (19,20). In particular: the segregation program of AB1157 appears to be significantly more simple.

Specifically, previous studies have reported that the nucleoid undergoes a number of large-scale structural rearrangements during the segregation process (19,20). These unsnapping events should lead to a number of clearly visible steps in the mean *oriC* trajectory if the unsnapping events occur at a synchronous time with respect to the splitting of the origin. We do not observe any clear step-like features in the mean trajectory above the noise background, suggesting that if these events do play a role in segregation in AB1157, the timing of these events is not coordinated with *oriC* splitting.

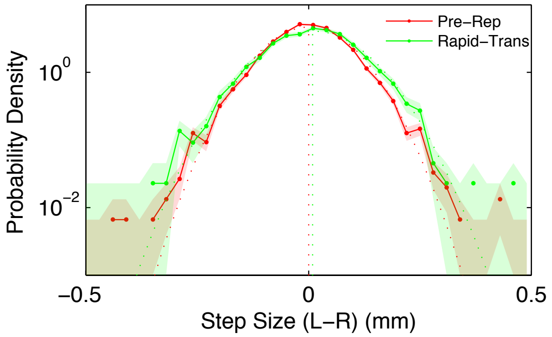

FIG. S5. Step size distribution for the pre-replication and rapid-translocation phases for cells with a cell cycle duration between 115-125 minutes (158 cells).

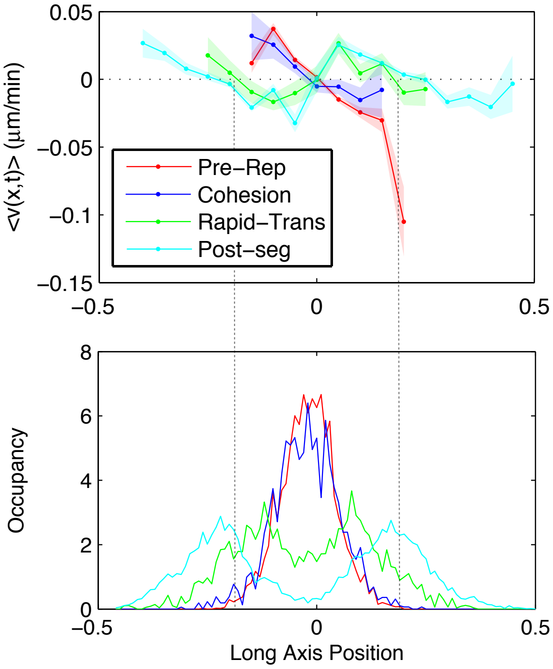

FIG. S6. Spatiotemporal drift velocity and occupancy for the four segregation phases for cells with a cell cycle duration between 115-125 minutes (158 cells).

S3. R1-16 PLASMID PARTITIONING

To be consistent with our chromosome measurements, for MSD analysis of R1-16 plasmid partitioning (as discussed in the main text) we chose cells that only exhibit a single segregation event, i.e. cells that begin the cell cycle with one fluorescent focus and divide with two. A typical kymograph of R1-16 replication is shown in Fig. S7.A. Figure S7.B shows MSD for the pre- and post-separation phases (we do not define the *Cohesion* phase for the plasmid). We see a MSD scaling parameter of 0.56±0.04, far lower than the ballistic scaling of 2.

This result does not imply that R1-16 plasmid partitioning is a non-active process, but rather that an active segregation mechanism can still display sub-diffusive dynamics on the length and time scale of the cell cycle. This apparent ambiguity exemplifies the limitations of MSD analysis in quantitatively characterizing the dynamics of sub-cellular bacterial components, and strongly motivates the deeper spatiotemporal analysis presented in the main text.

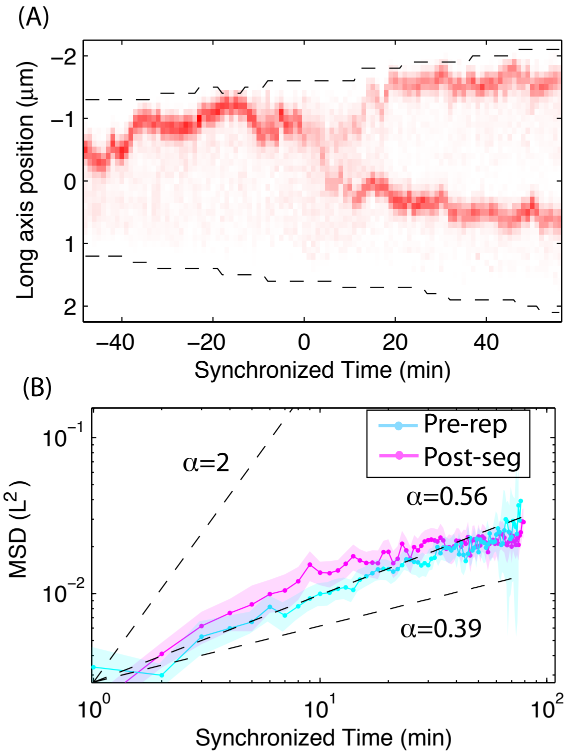

FIG. S7. Mean-Squared Displacement (MSD) for the actively partitioned R1-16 plasmid. Even though the Type II partitioning system of the plasmid is known to be an active process, the dynamics of R1-16 prior to and following separation are actually sub-diffusive, signified by an MSD scaling parameter $\alpha = 0.56$. This result implies that an active segregation mechanism, which must appear ballistic on long enough time scales, can appear sub-diffusive on the length scale of the cell cycle.

S4. SPATIOTEMPORAL DYNAMICS

Along with the mean drift velocity profile shown in the main text, we can also calculate other dynamics properties of our *oriC* trajectories. Figure S8 shows the single step velocity autocorrelation, $\langle \delta x(t) \delta x(t+1) \rangle$, of successive steps during the four phases of chromosome segregation. In all phases, the autocorrelation is negative and relatively flat, consistent with the previously discussed viscoelastic model of chromosomal loci (25). Figure S9 shows the effective diffusion constant, defined as $\left\langle \delta x^2(x,t) \right\rangle$. There is a slight increase (50%) in mobility during the *Cohesion* and *Rapid Translocation* phases, possibly as a result of local de-condensation event of regions of the chromosome near *oriC*.

S5. PUTATIVE CENTROMERIC SITE *MIGS*

To investigate whether or not *migS* is the source of force generation during the segregation process, we transduced *yijF*::KmR from the KEIO collection into our two-color AB1157 strain using P1, and verified the deletion by colony PCR (32). If *migS* is indeed a centromeric-like site, we would expect to not observe the immediate shift in the equilibria positions in the drift velocity profile following *oriC* splitting. The spatiotemporal drift velocity profile and occupancy for 341 cells is shown in Fig. S10. The drift velocity profile retains the rapid shift in equilibria position, suggesting that *migS* is not the source of force generation during the segregation process.

S6. VISUALIZATION PLASMID

For all data presented here and in the main text we used a combination of FROS (fluorescent repressor operator system) and the ParB-*parS* system to visualize specific loci on the chromosome. Strain details are given in the main text, and expression plasmid map is shown in Fig. S11.

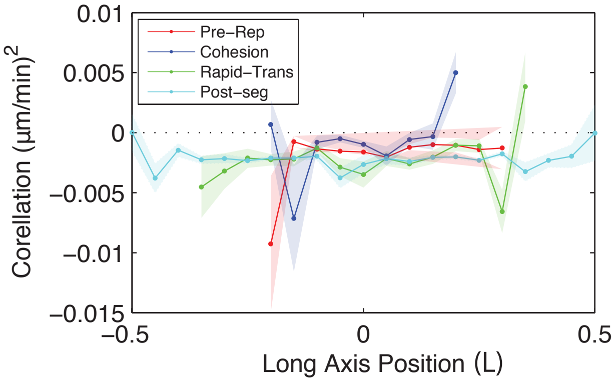

FIG. S8. Mean velocity autocorrelation of successive steps in *oriC* trajectories as a function of spatial location in the cell. The mean autocorrelation in all four phases of the cell cycle is negative and essentially flat, consistent with the previously reported viscoelastic memory properties of chromosomal motion.

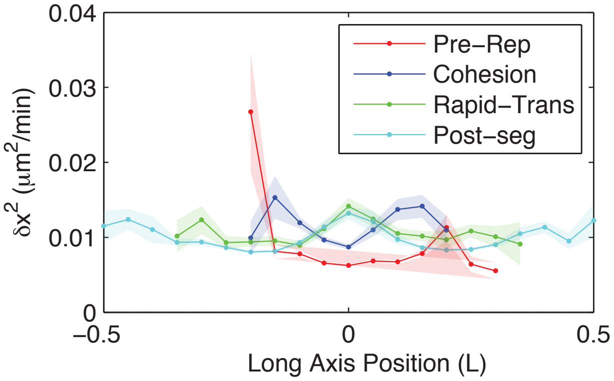

FIG. S9. Effective diffusion constant of *oriC* as a function of spatial location in the cell, defined as $D_{\text{eff}}(x,t) = \langle \delta x^2(x,t) \rangle$ as a function of spatial location in the cell. There is a slight increase in the effective diffusion constant ($\sim$ 50%) in the *cohesion phase*, consistent with local de-condensation of the surrounding nucleoid.

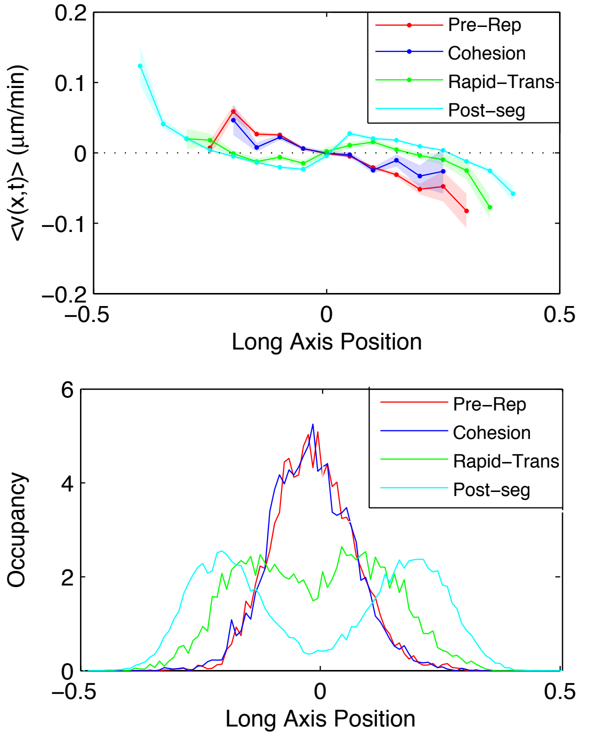

FIG. S10. Spatiotemporal drift velocity profile (top) and occupancy (bottom) of *oriC* in cells lacking the putative centromeric site *migS*. The drift velocity profile retains the same immediate shift in equilibria positions following the split, suggesting that *migS* is not the origin of segregation forces.

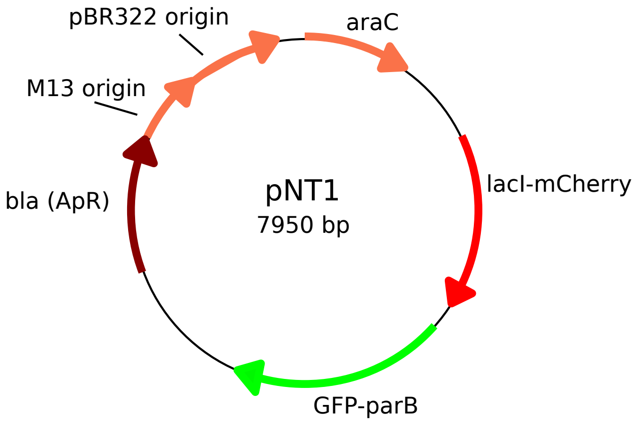

FIG. S11. Two color expression plasmid pNT1. To ensure faithful inheritance of the expression plasmid, cells were grown on LB-agar plates and in liquid cultures inoculated with 100 $\mu g/mL$ ampicillin. For microscopy, cells were induced with 0.02% l-arabinose for 30 minutes at 30°.
